# Effect of a breathing mask on type 2 mediator responses to subzero exercise in individuals without asthma

**DOI:** 10.1186/s13104-026-08094-w

**Published:** 2026-10-28

**Authors:** Helen Hanstock, Mats Ainegren, Erik P. Andersson, Ella Bäckebjörk, Nikolai Stenfors

**Affiliations:** 1 Swedish Winter Sports Research Centre, Department of Health Sciences, Mid Sweden University, Östersund, Sweden; 2 Department of Engineering, Mathematics, and Science Education, SportsTech Research Centre, Mid Sweden University, Östersund, Sweden; 3 Department of Public Health and Clinical Medicine, Umeå University, Umeå, Sweden

**Keywords:** Cold environment, Exercise-induced asthma, Exercise-induced bronchoconstriction, Occupational health, Winter sports

## Abstract

**Objective:**

Current evidence suggests that heat and moisture-exchanging breathing masks (HME) protect against exercise-induced bronchoconstriction (EIB). We aimed to explore whether mast cell mediator release following exercise in subzero conditions is affected by the use of an HME. To this end, 23 healthy, recreational-competitive endurance athletes aged 18–53 years without asthma performed a 30-min warm-up followed by a 4-min maximal, self-paced running time trial, with and without HME, in -15 °C. Concentrations of leukotriene E_4_ (LTE₄) and 11β-prostaglandin F2α (11β-PGF2α) were measured in urine pre- and 60 min post-exercise and data analysed using repeated-measures ANOVA.

**Results:**

LTE₄ decreased after the 4-min maximal exercise test (*p* < 0.001), with no significant interaction with HME use. There was no effect of exercise alone nor HME on 11β-PGF2α responses to exercise. Taken together, we observed limited evidence of type 2 inflammation in healthy participants after brief, high-intensity exercise in the cold, with and without use of an HME. As the biomarker outcomes reported here were secondary and not powered in advance, these exploratory findings in a healthy cohort warrant further investigation using more robust airway challenges to better understand early airway responses and the potential for preventative strategies in athletes.

**Trial registration:**

ISRCTN, Registered 01 April 2019, https//doi.org/10.1186/ISRCTN10018222.

## Introduction

Elite winter endurance athletes have a high prevalence of lower airway dysfunction, including asthma and exercise-induced bronchoconstriction (EIB) [1]. Unlike allergic asthma, which often begins earlier, asthma and EIB in athletes tend to present during early adolescence [2], at a time that may coincide with increased sports specialisation. Whereas the pathophysiological mechanisms of allergic asthma, characterised by eosinophilic (type 2) airway inflammation, have been thoroughly studied, the pathogenesis of lower airway dysfunction in otherwise healthy athletes remains poorly understood [3]. This latter group has been hypothesized to typically present with non-allergic phenotype and non-type 2 asthma endotype [4], but the role of type 2 inflammation in the development of EIB in athletes remains unclear.

We recently demonstrated that use of a heat- and moisture-exchanging breathing mask (HME) by healthy individuals during exercise in cold air attenuated decreases in lung function, markers of airway epithelial damage and airway symptoms post-exercise, compared to exercising without an HME [5]. As an exploratory extension of the aforementioned study, our objectives here were to examine whether exercise in a subzero environment elicited a type 2 inflammatory response in healthy subjects and to assess whether HME usage can affect these responses. We selected urinary leukotriene E_4_ (LTE_4_) and 11β-prostaglandin F2α (11β-PGF2α) as outcome measures because they can be measured non-invasively as stable end-metabolites of mast cell activation, the effector pathway most directly implicated in the acute airway response to hyperpnoea, and because both have previously been shown to increase after dry air hyperpnoea in athletes [9].

To this end, 23 recreational-competitive level athletes (15 male, 8 female), aged 31 ± 8 years (mean ± SD), without asthma, provided written informed consent to participate in the study, which was approved by the Umeå Regional Ethical Review Board (Dnr: 2018–419 − 31) and conducted in accordance with the Declaration of Helsinki. Participants performed two exercise sessions in an environmental chamber set to -15 °C, with or without HME, in a randomised, cross-over design [5]. Trial order was block randomised in groups of 4 on a rolling basis in order of enrollment. The random sequence, enrollment, and allocation of trials were performed by separate members of the research team, and personnel obtaining and analysing samples (but not participants) were blinded to the intervention. Participants provided urine samples before and 60 min after each exercise session which were assessed for two biomarkers, LTE_4_ as a marker of cysteinyl leukotriene release in the lungs and 11β-PGF2α as the primary urinary metabolite of prostaglandin D2, both of which have been associated with type 2 asthma [6]. These biomarkers were analysed using enzyme immunoassay kits (detection limits 25 pg/ml and 5 pg/ml, respectively) from Cayman Chemical (Ann Arbor, MI, USA). One sample was missing (post-HME trial). All samples were run in duplicate with an intra-sample variation of < 5%. Concentrations of LTE_4_ and 11β-PGF2α are reported relative to creatinine concentrations. The residuals were checked for normality and data were log_10_ transformed before analysis. We used a two-way repeated-measures ANOVA with Bonferroni post-hoc tests to analyse differences in exercise responses between trials. The outcome variables described within the present study were not primary outcomes and thus no a priori sample size calculation was performed. The study is therefore likely to be underpowered to detect small between-trial differences in biomarker responses.

**Fig. 1:**
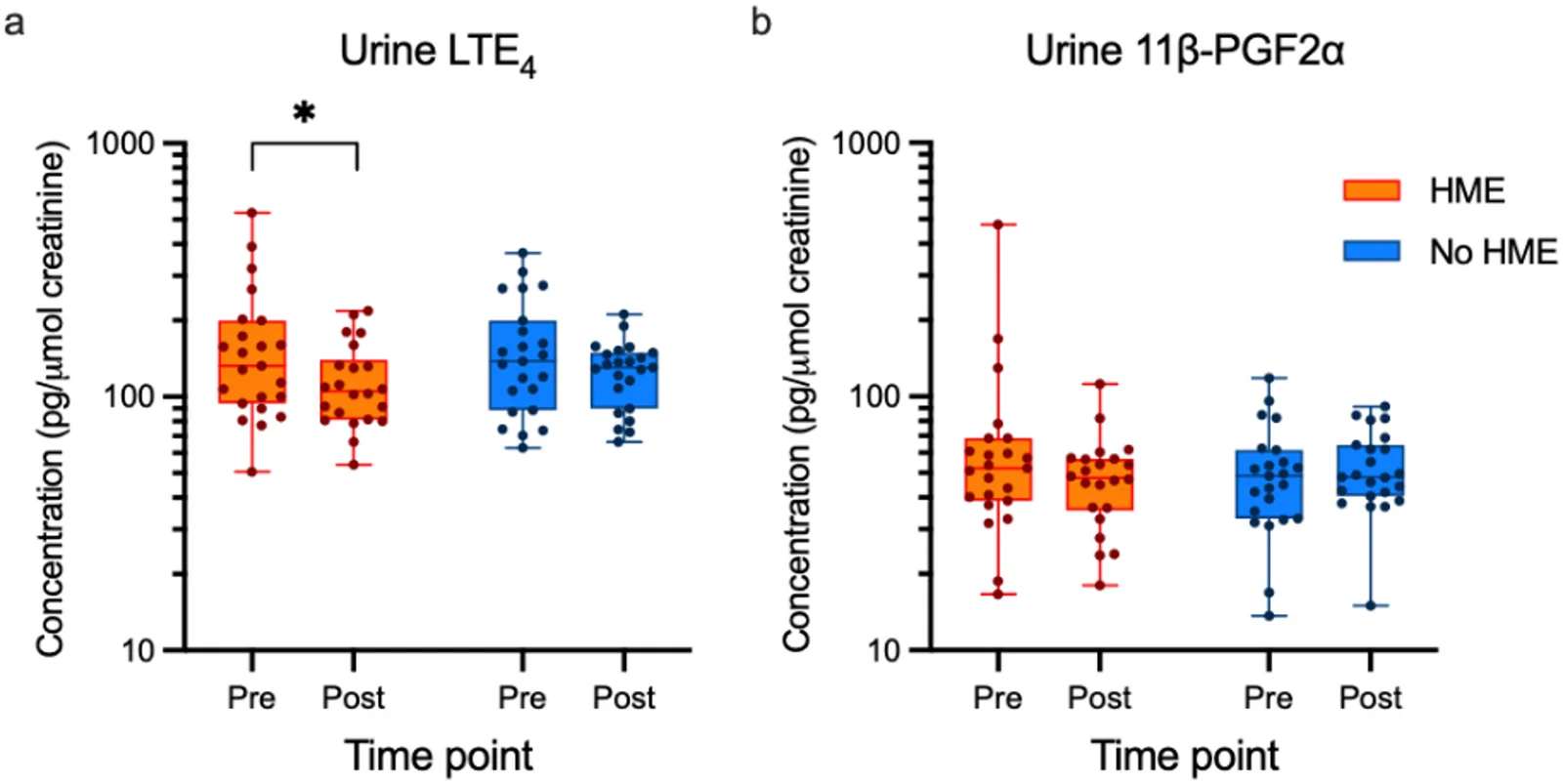
LTE_4_ (a) and 11β-PGF2α (b) responses following maximal exercise in − 15 °C with and without HME. Boxes show min to max with all individual data points, with line drawn at the median. *, difference between pre and 1-hour post-exercise, *p* < 0.05

LTE_4_ decreased from pre- to post-exercise (main effect of time, F(1, 21) = 15.8, *p* < 0.001, η²_p_ = 0.43). Estimated marginal means (log_10_ scale) decreased from 2.15 [95% CI: 2.07–2.23] pre-exercise to 2.06 [2.01–2.11] post-exercise. Although there was no significant time × trial interaction (F(1, 21) = 0.51, *p* = 0.49, η²_p_ = 0.02), exploratory post-hoc tests indicated a significant post-exercise decrease in LTE_4_ for the HME trial (*p* = 0.042), but not for the trial without HME (*p* = 0.64; Fig. 1a). In the absence of a significant interaction effect, this post-hoc difference should not be taken as evidence of a differential response between trials. 11β-PGF2α did not respond to exercise, irrespective of trial (interaction effect: F(1, 21) = 1.52, *p* = 0.23, η²_p_ = 0.07; main effect of time: F(1, 21) = 0.22, *p* = 0.64, η²_p_ = 0.01; Fig. 1b).

Development of EIB in both asthma and in athletes without pre-existing airways disease is generally attributed to the combination of high ventilation with repeated exposure to environmental stressors, such as allergens, air pollutants, or dry and/or cool air [7]. Exercise-induced hyperpnoea causes shear stress and microtrauma to the airway epithelium, potentially triggering the activation of eosinophils and mast cells. The subsequent release of histamine, prostaglandin D_2_, and cysteinyl leukotrienes, then contributes to vascular leakage, oedema, and smooth muscle contraction [8]. This process is likely exacerbated in cold environments, where dry air accelerates water loss from the airway mucosa, increasing osmolarity in epithelial cells and causing cell shrinkage and damage [7].

The findings from our analysis should be interpreted with caution. On one hand, LTE_4_ decreased after exercise and 11β-PGF2α remained unchanged, whereas we might have expected an increase in these biomarkers, as has been reported following dry air hyperpnoea in athletes with, and to a lesser extent without, EIB [9]. However, in the present study, the average decrease in forced expiratory volume in 1s (FEV_1_) after exercise was only 2.7% without HME and 0.5% with HME [5]. Although this difference was sufficient to reveal a significant effect of trial, the magnitude of the FEV_1_ response in both trials was substantially below the 10% threshold used to define EIB. The magnitude of the airway response may therefore have been unlikely to provoke substantial type 2 inflammatory responses in our cohort. However, the focus on healthy individuals was intentional, as the parent study aimed to investigate possible bronchoprotective effects of HMEs after cold-weather exercise with an ultimate focus on the potential for primary prevention of EIB, whereas the protective effects of HME in individuals with established EIB are better described [10–12].

Although the present study is unable to provide direct evidence that HME use altered type 2 inflammatory mediators, the direction of the LTE_4_ response following exercise with HME is not inconsistent with previous reports of a potential protective effect of the HME on the airway epithelium and submucosa in healthy individuals [5] and may warrant further investigation. It remains possible that an attenuation of epithelial stress could evoke differences in inflammatory signalling after exercise in cold environments, which could represent one mechanism by which an HME can attenuate EIB in individuals with asthma [13]. However, this hypothesis requires confirmation in adequately powered studies specifically designed to address this question.

Further research is needed to better understand the effect of training and environmental factors on inflammatory airway responses in healthy individuals, as well as to investigate potential countermeasures to prevent the development of lower airway dysfunction in winter athletes. Taken together, the present study does not provide clear evidence for an effect of HME use on type 2 inflammatory responses following brief, high-intensity cold-weather exercise. Given the modest FEV_1_ response observed in this cohort, the exercise challenge was unlikely to have elicited a measurable type 2 inflammatory response, and the absence of biomarker changes should therefore not be interpreted as evidence that airway inflammation did not occur. These exploratory findings apply only to healthy, recreational-competitive endurance athletes under the specific experimental conditions employed and should not be extrapolated to athletes with asthma or EIB. The observed trends warrant further investigation in adequately powered studies, incorporating more robust airway challenges and broader inflammatory profiling across populations with and without lower airway dysfunction.

## Limitations

This study recruited only healthy individuals, without including comparison groups of individuals with lower airway dysfunction, whether of an atopic or sports endotype [14], so we have no reference values for a type 2 response in athletes with lower airway dysfunction after this specific exercise protocol. In addition, we did not consider whether our participants were atopic. Since atopy influences both baseline and stimulated release of cysteinyl leukotrienes and prostaglandin D_2_, the unknown atopic status of our participants limits the interpretation of the biomarker data. The observations reported here therefore have limited translational value at present to athletes with lower airway dysfunction, although they may inform researchers as to whether to account for these pathways when studying acute effects of dry air hyperpnoea on the airways of healthy individuals. Additionally, only two biomarkers of type 2 inflammation were assessed at a single post-exercise time point, which could provide a limited view of the post-exercise inflammatory response, and it is possible that peak mediator release could have occurred outside the sampling window; for example, previous work has reported the peak mediator concentration at any time-point between 30 and 90 min [9]. In particular, the post-hoc test showing a significant decrease in LTE_4_ after exercise with HME is not supported by a significant interaction effect, which limits the strength of inference that can be drawn from this exploratory observation, which could represent a true physiological effect, biological variability, or a chance finding. Finally, no a priori sample size calculation was performed for the included outcomes in the parent study, and thus the absence of measurable biomarker responses should not be interpreted as evidence that airway inflammation did not occur, but rather that any response may have been below the level detectable under the conditions of the present study.

## Data Availability

All data supporting the findings of this study are available within the paper.

## Declarations

Participants provided written informed consent to participate in the study, which was approved by the Umeå Regional Ethical Review Board (Dnr: 2018–419 − 31) and conducted in accordance with the Declaration of Helsinki.

## Abbreviations

11β: PGF2α− 11β prostaglandin F2α
AHR: airway hyperresponsiveness
ANOVA: analysis of variance
EIB: exercise-induced bronchoconstriction
FEV_1_: Forced expiratory volume in one second
HME: heat-and moisture-exchanging breathing mask
LTE_4_: leukotriene E_4_
SD: standard deviation
